# An Experiment with Forced Oxygenation of the Deepwater of the Anoxic By Fjord, Western Sweden

**DOI:** 10.1007/s13280-014-0524-9

**Published:** 2014-05-01

**Authors:** Anders Stigebrandt, Bengt Liljebladh, Loreto de Brabandere, Michael Forth, Åke Granmo, Per Hall, Jonatan Hammar, Daniel Hansson, Mikhail Kononets, Marina Magnusson, Fredrik Norén, Lars Rahm, Alexander H. Treusch, Lena Viktorsson

**Affiliations:** 1 Department of Geosciences, University of Gothenburg, Box 460, 405 30 Göteborg, Sweden; 2 Analytical and Environmental Chemistry, Vrije Universiteit Brussel, Pleinlaan 2, 1050 Brussels, Belgium; 3 Nordic Center for Earth Evolution Institute of Biology, University of Southern Denmark, 5230 Odense M, Denmark; 4 Marine Monitoring AB, Strandvägen 9, 453 30 Lysekil, Sweden; 5 Department of Chemistry and Molecular Biology Marine Chemistry, University of Gothenburg, 412 96 Göteborg, Sweden; 6 Swedish Institute for the Marine Environment, Box 260, 405 30 Göteborg, Sweden; 7 Department of Chemistry and Molecular Biology, Marine Chemistry, University of Gothenburg, 412 96 Göteborg, Sweden; 8 IVL Swedish Environmental Research Institute, Rosviksgatan 12, 45330 Lysekil, Sweden; 9 Department of Thematic Studies, Water and Environmental Studies, Linköping University, 581 83 Linköping, Sweden

**Keywords:** Deepwater oxygenation, Environmental engineering, Anoxia, Ecological effects, Sediment colonization, Phosphorus

## Abstract

In a 2.5-year-long environmental engineering experiment in the By Fjord, surface water was pumped into the deepwater where the frequency of deepwater renewals increased by a factor of 10. During the experiment, the deepwater became long-term oxic, and nitrate became the dominating dissolved inorganic nitrogen component. The amount of phosphate in the water column decreased by a factor of 5 due to the increase in flushing and reduction in the leakage of phosphate from the sediments when the sediment surface became oxidized. Oxygenation of the sediments did not increase the leakage of toxic metals and organic pollutants. The bacterial community was the first to show changes after the oxygenation, with aerobic bacteria also thriving in the deepwater. The earlier azoic deepwater bottom sediments were colonized by animals. No structural difference between the phytoplankton communities in the By Fjord and the adjacent Havsten Fjord, with oxygenated deepwater, could be detected during the experiment.

**Electronic supplementary material:**

The online version of this article (doi:10.1007/s13280-014-0524-9) contains supplementary material, which is available to authorized users.

## Introduction

Coastal areas occupied by hypoxic and anoxic waters are increasing globally (Diaz and Rosenberg 2008). In the Baltic Sea, expanding hypoxia and anoxia decreases ecosystem services and changes the retention of nutrients (e.g., Carstensen et al. 2014). At present, the Baltic proper experiences huge internal leakage of phosphorus from anoxic bottoms in the Baltic proper (e.g., Stigebrandt et al. 2013; Viktorsson et al. 2013a) and increased nitrogen–phosphorus imbalance even in the surface layers, leading to vast summertime blooms of cyano bacteria. The occurrence or not of anoxia in the deepwater of semienclosed basins depends on the rates of supply of oxygen and organic matter, respectively. It has been suggested that environmental engineering methods may be used to increase the rate of oxygen supply to deep anoxic basins, e.g., by Stigebrandt and Gustafsson (2007) who discussed large-scale oxygenation of the deepwater to reduce eutrophication of the Baltic proper. A review of engineering methods and possible consequences of applying them on the Baltic Sea is given by Conley et al. (2009). Several authors call for caution and discuss various possible negative consequences of man-made oxygenation, e.g., leakage of toxic substances from earlier anoxic bottoms due to deep-digging worms (Conley 2012), and building up large amounts of iron-bound phosphorus in oxygenated sediments that may be released when the oxygenation is shut off (Reed et al. 2011). An example of the latter can be seen in the Bornholm Basin where the deepest bottoms since the 1960s switch between anoxic and oxic states (Stigebrandt et al. 2013).

Ideally, environmental consequences of long-term oxygenation of anoxic basins may be studied in basins naturally switching between oxic and anoxic states. However, there are a few basins where the oxic periods are long enough for long-term changes to occur. Therefore, the most environmental effects of long-term oxygenation seem to be uncertain, which leads to speculation. To decrease the uncertainty about environmental effects and costs, it is needed to make adequate experiments of sufficient duration to learn more about environmental impacts of oxygenation of anoxic basins. The Baltic deepwater oxygenation (BOX) project made a pilot experiment in the By Fjord where most of the usually anoxic deepwater was kept oxic for a duration of about 2.5 years by pumping oxygen-rich surface water into the deepwater. The present paper provides an overview of the experiment including the pump system, costs to pump, and the observed environmental impact.

## Materials and Methods

### Location, Hydrography, and Water Quality

**Fig. 1:**
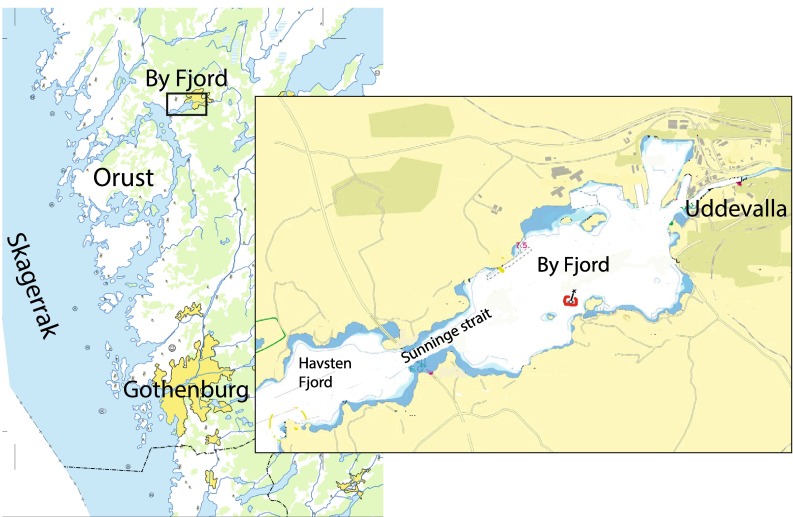
Map over the area. The *red circle* indicates the position of the pump station

The By Fjord on the Swedish west coast is the innermost basin in the fjord system between the Island Orust and the mainland (Fig. 1). The Sunninge Strait (sill depth 13 m) connects the By Fjord with the Havsten Fjord. The horizontal surface area of By Fjord equals 6.15 km^2^, and the largest depth is about 50 m. The volumes above and below sill depth are 0.065 and 0.073 km^3^, respectively. The dominating tide is semi-diurnal with a range of only 0.3 m. The fjord is strongly stratified with a halocline just below sill depth. The water exchange is mainly forced by density variations in the coastal water, leading to a residence time of about 1 week above the sill but up to 3–5 years below 20-m depth (e.g., Hansson et al. 2012). The long residence time of the deepwater, below the sill, is due to the combined effect of very weak vertical mixing in the fjord basin and large subannual density variations outside the fjord—see Stigebrandt and Liljebladh (2011). A surface layer with reduced salinity develops in periods of large local freshwater supply.

The Bäve River, debouching in the city of Uddevalla with about 50 000 inhabitants, is the largest river in the fjord system with an annual mean flow of about 8 m^3^ s^−1^. It annually transports roughly 9 tonnes of phosphorus and 165 tonnes of nitrogen to the fjord. Uddevalla sewage treatment plant, Skansverket, introduced reduction of phosphorus in 1974 and nitrogen in 1994. Its annual outputs, about 2 tonnes of phosphorus and 50 tonnes of nitrogen, are discharged into the mouth of Bäve River (Viktorsson 2007). Total iron and manganese loads are 110 and 11 tonnes year^−1^, respectively (http://www.slu.se/sv/miljoanalys/statistik-och-miljodata/).

The rapid water exchange above the sill leads to usually good water quality in these layers, while the very long residence time of deepwater below 20-m depth leads to accumulation of hydrogen sulfide, phosphate, and ammonium. The layer between 13 and 20 m is periodically anoxic with the occurrence of hydrogen sulfide. The environmental status of the fjord can be described using a benthic habitat quality index (BHQ). Index gradients due to oxygen gradients are explained in Nilsson and Rosenberg (2000). Above the sill, the BHQ in the By Fjord is almost as high as in the oxygenated Havsten Fjord. Beneath the sill, however, BHQ rapidly decreases to zero because oxygen is usually absent. During deepwater renewals in By Fjord, some of the old deepwater is mixed (entrained) into the new deepwater, while the remainder with its contents of hydrogen sulfide, ammonium, and phosphate, is lifted above the sill and flushed out to the upper layers of Havsten Fjord where it perhaps might cause eutrophication and other ecological stresses.

### The Pump System

A system for forced oxygenation of the deepwater in the By Fjord was constructed. It pumps oxygen-rich surface water of often low salinity into the deepwater, which strongly increases the rate of density reduction of the deepwater, and thereby the frequency of deepwater renewals by inflows of dense water from the adjacent Havsten Fjord. The pumping thus brings oxygen to the deepwater in two ways: by adding oxygen-rich surface water, and by inducing more frequent deepwater renewals.

**Fig. 2:**
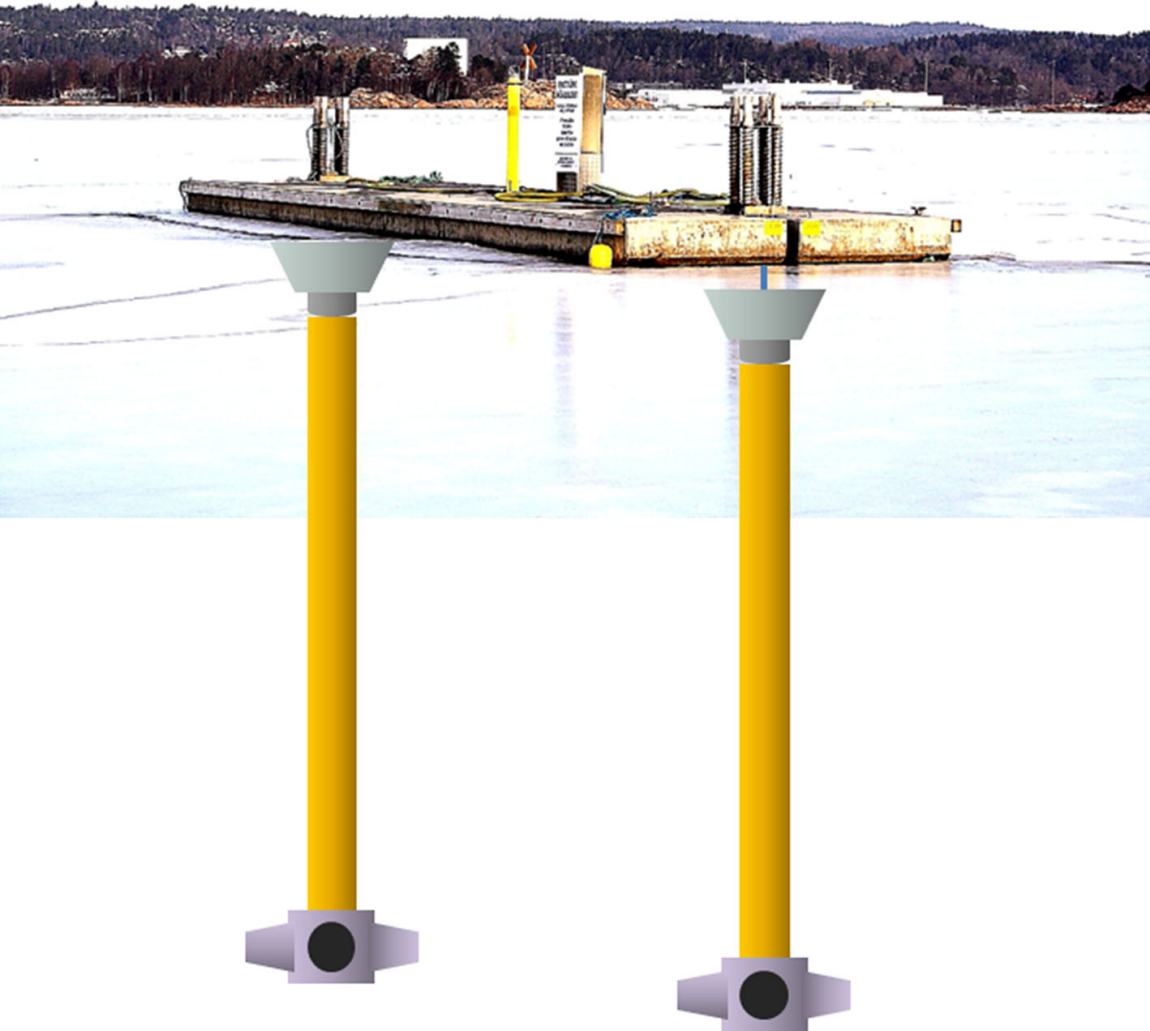
The pumps hang inside the intake cones in feather clutches below a floating concrete jetty and the nozzles hang in fabric pipes

From model calculations using historic hydrographical data, it was concluded that pumping 2 m^3^ s^−1^ would be sufficient to keep the deepwater oxic (Stigebrandt and Liljebladh 2011). To ease the mechanical handling of the system, it was decided to use two electrically powered units, each having a nominal pumping capacity of 1 m^3^ s^−1^. The fabric pipes are 32 m in length with a diameter of 0.8 m (Fig. 2). The water is ejected from the nozzles, at about 35-m depth which is about 6 m above the sea bed, as horizontal jets that after a couple of meters of intense mixing transform to become buoyant plumes. The speed of the water through a pipe and its four nozzles equals 2 m s^−1^. The intake cone, with the base reaching about 2 m below sea surface, was constructed to ensure that water is (selectively) withdrawn horizontally into the cone. Flow estimates, from observations of travel time of a tracer through the pump system and direct measurements of speed at the nozzles, indicate that the actual pumped flow rate is in the range of 1.8–2.1 m^3^ s^−1^. From October 2010 to December 2012 the pumps were on duty for about 12 000 h transferring about 86 million cubic meters of surface water to the outlets. Cost estimates of pumping in the By Fjord are presented in Electronic Supplementary Material.

### Observational Program and Methods of Analyses

During the experiment, the established monitoring program in the By and Havsten Fjords was intensified from monthly to bimonthly. The program included sampling of temperature, salinity, dissolved oxygen, hydrogen sulfide, nutrient concentrations (PO_4_, Total P, NO_2_, NO_3_, NH_4_, Total N, and Si(OH)_4_), chlorophyll-a, particulate organic carbon, and particulate organic phosphorus (POC and POP) at the standard depths (0, 2, 5, 10, 15, 20, 30, 40, and just above the greatest depth). Methods of analyses are given in Electronic Supplementary Material. Phytoplankton was sampled over two integrated depth intervals (0–10 and 10–20 m), and the methods of analyses are described in Norén (2012).

A mooring with several Seabird loggers and AADI oxygen optodes monitored in almost real-time state of the fjord in terms of salinity, temperature, and oxygen during the experiment. The loggers were wired to a control unit ashore with a 3G link to our web server. In addition, an acoustic current profiler (Teledyne) was deployed on the Sunninge Strait sill (13 m) also with a real-time link.

A special monitoring program for the experiment, ordered as a condition for carrying out the experiment, measured any changes in the benthic fauna community and environmental impact of pollution expressed as bioaccumulation of contaminants in caged deployed common mussels (*Mytilus edulis* L.) (Nelson 1990; Granmo 2004; Bellas et al. 2007; Magnusson et al. 2011). In addition, passive samplers, SPMD-semipermeable membrane devices (http://www.est-lab.com), and DGT—diffusive gels in thin films (Davison and Zhang 1994) were deployed in the anoxic deepwater 2–4 m above the bottom before start and during the oxygenation process. In total, 20 different metals and a range of organic pollutants were analyzed (listed in Table S3 in Electronic Supplementary Material). All analyses were performed by accredited laboratories using well-known methods (GC–MS, GC–FPD, ICP-AES, and ICP-SFMS).

Sediment profile imagery (SPI) was used to study changes of the marine benthic habitats. Sampling was conducted in the years 2009, 2011, and 2012, by deploying the sediment profile camera at 12 stations (with four replicates) that were divided equally in three depth strata: 10–16 m, 16–22 m, and below 22 m. The prism penetrates into the sediment like an inverted periscope and takes a 16 × 24 cm^2^ in situ two-dimensional picture of the sediment. Assessment of the benthic habitat quality index (BHQ) was calculated as described in Nilsson and Rosenberg (1997). The index is based on parameterization of biogenic structures caused by activities of the fauna on the sediment’s surface, within the sediment, and the mean vertical depth distribution of the apparent redox potential discontinuity (aRPD).

The study of the benthic faunal community and possible vertical colonization due to the forced oxygenation of the fjord was divided into two depth intervals (10–20 m and below 20 m) and took place during four sampling occasions: May 2009, May 2011, May 2012, and October 2012. On each sampling occasion, six grab samples were taken between 10 and 20 m and below 20 m, respectively, at random locations. The fauna was sampled with a 0.1 m^2^ Smith-McIntyre grab, and the samples were sieved through 1-mm meshes.

Changes in bacterial community structure were monitored by terminal restriction fragment length polymorphism (T-RFLP) analysis and clone libraries of the 16S rRNA gene. A total of 40 samples from above, within, and below the oxycline of the By Fjord, collected during eight cruises from 2010 to 2012, were analyzed. For comparison, eight samples from Havsten Fjord and seven samples from Koljö Fjord (north of Orust Island) were analyzed as well. Resulting bacterial community fingerprints were statistically analyzed using nonmetric methods and put into the context of environmental parameters (Forth et al., in preparation).

Benthic fluxes of dissolved inorganic phosphorus (DIP) were measured in situ using one or two of the Gothenburg benthic chamber landers (e.g., Ståhl et al. 2004). Flux measurements were made on permanently oxic as well as on originally anoxic stations before and after forced oxygenation of the deepwater. Incubations and analyses were performed as described in Viktorsson et al. (2013b).

To test the classic paradigm of PO_4_ retention by binding to Fe and Mn oxides in oxic environments, and also the reversal as the latter goes anoxic and the metals dissolve again (Mortimer 1941), Fe and Mn in the water column were sampled using cation DGT (Davison and Zhang 1994), and analyzing them by the USEPA methods 200.7 (ICP-AES) and 200.8 (ICP-SFMS). The sampling in the vicinity of the pumping site comprised 17 periods during two and a half years, covering 79 % of the test period. Usually, seven depths were sampled in the deepwater.

## Results

### Fjord Circulation and Effects of Pumping Down Surface Water

**Fig. 3:**
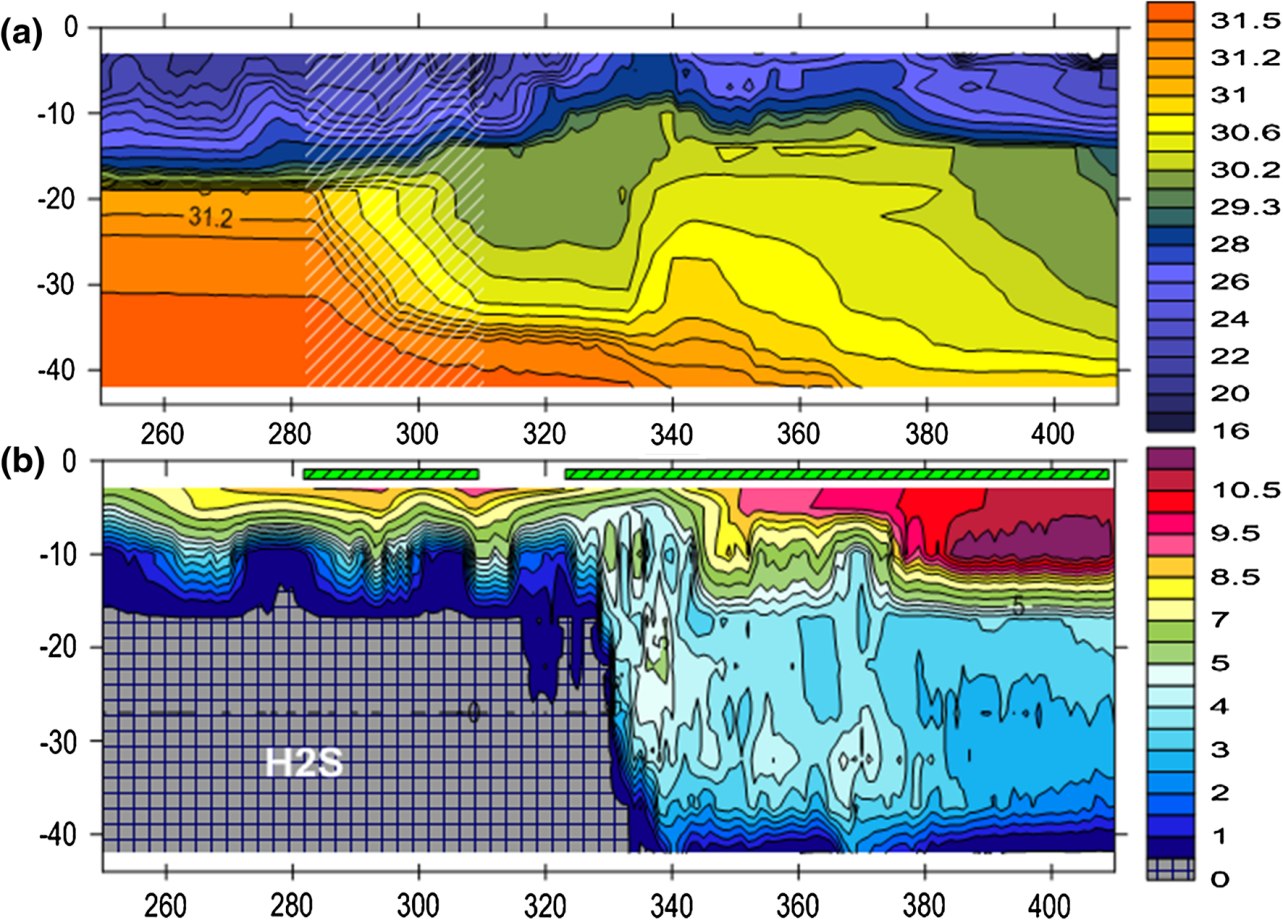
Salinity (**a**) and dissolved oxygen mg L^−1^ (**b**) from the mooring in By Fjord from Sep 7, 2010 (day no 250) to Feb 14, 2011 (day no 410) (horizontal axis). The highlighted area in (**a**) shows the salinity reduction during the first pump period. The pumps were on as indicated by the green rectangles on top of (**b**). 12 CTD—7 of these with oxygen sensors—were mounted on the mooring during the time displayed

After having corrected some initial defects of the pump equipment, pumping started on Oct 8, 2010 (day number 282). Before this day, contour lines of, e.g., salinity were almost horizontal in the deepwater due to very weak natural vertical mixing (Fig. 3a). During pumping, contour lines in the deepwater sank by about 2 m day^−1^ due to entrainment of ambient water into the pumped jets and rising buoyant plumes, see the highlighted part in Fig. 3a. Since the horizontal area of the fjord in the deepwater is typically 2.5 × 10^6^ m^2^, the vertical upward transport of entrained water by the plumes equals about 5 × 10^6^ m^3^ day^−1^, which is a factor 30 larger than the pumped volume (1.7 × 10^5^ m^3^ day^−1^). The turnover time of the pump affected volume (~50 × 10^6^ m^3^) was thus around 10 days. The decrease in the rate of salinity of the deepwater due to dilution by surface water was in the range 0.025–0.04 day^−1^, meaning that it took 25–40 days to decrease the salinity by one unit. This is two orders of magnitude greater than the rate driven by natural vertical mixing. CTD-casts evenly distributed over the fjord showed that although pumping is undertaken in only one position, the result of the pumping is evenly distributed over the whole basin due to horizontal currents that partly are induced by the pumping.

**Fig. 4:**
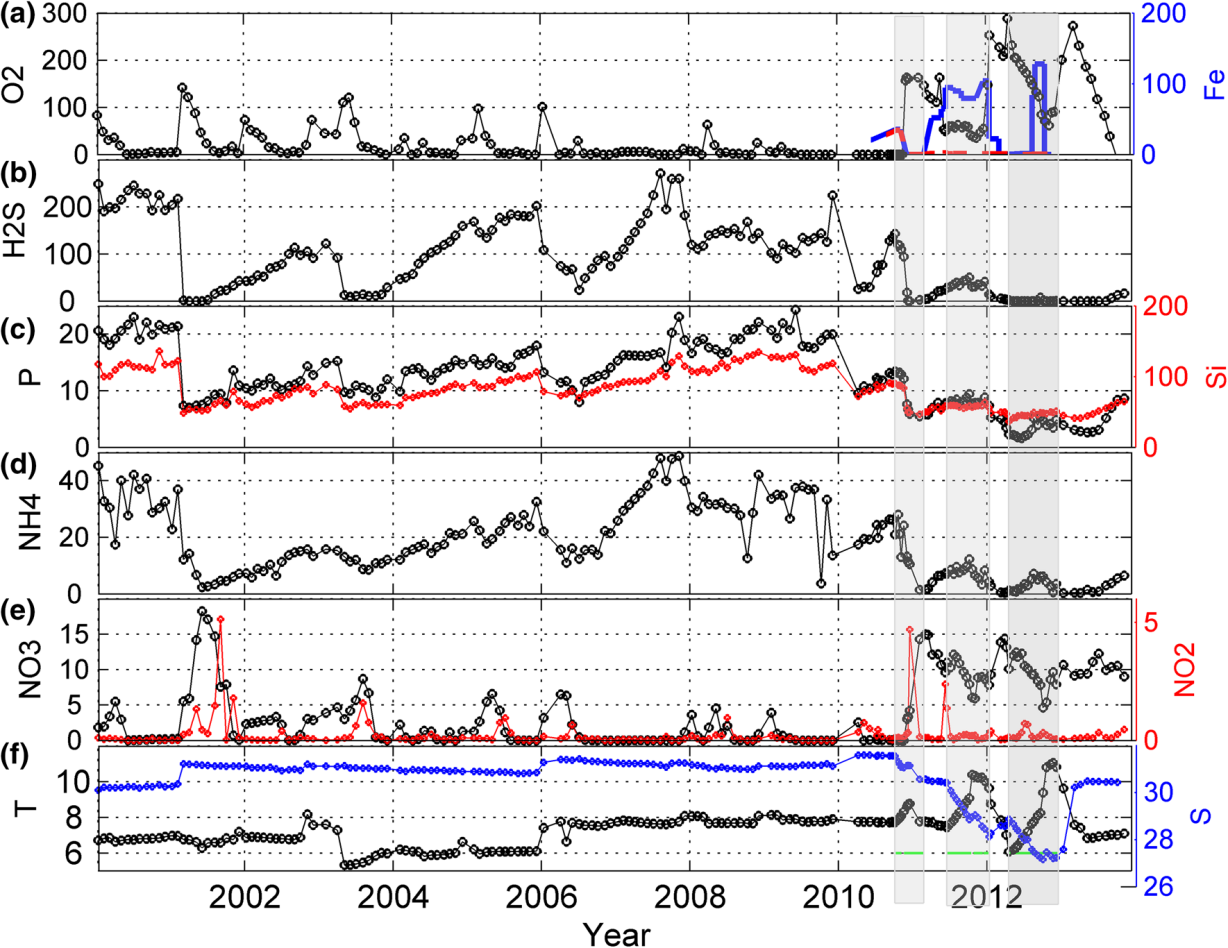
Panels **a**–**e** show total amounts (in tonnes) in the volume below 17.5 m from 2000 to 2014 of **a** Oxygen, **b** Hydrogen Sulfide, **c** Phosphate and Silicate (*red*), **d** Ammonium, and **e** Nitrite (*red*) and Nitrate. Panel **f** shows the volume mean Temperature, and the volume mean Salinity (*blue*). **a** Shows the concentration of Fe^2+^ (in µg L^−1^), at 32 (*blue*) and 41-m depth (*red dashed*), respectively. The *gray* shaded areas show the periods when pumping was on

The first water exchange came in late November 2010 (day 335). It is seen as rapidly rising contour lines (Fig. 3a). Estimates using observations in Havsten Fjord showed that inflowing new deepwater and old deepwater were mixed in about equal proportions. The deepwater became oxygenated because the new deepwater contained more oxygen than that was needed to oxidize the hydrogen sulfide (H_2_S) and ammonium (NH_4_) from the old deepwater which was mixed into the new deepwater (Fig. 3b). Before the water exchange, about 70 tonnes of H_2_S, 25 tonnes of NH_4_, and 11 tonnes of phosphate (PO_4_) were present below 17.5 m—see Fig. 4 that shows the development of the stock of nutrients, oxygen, H_2_S, and volume means of temperature and salinity since year 2000 in the volume below 17.5 m (~58 × 10^6^ m^3^). After the exchange, the entire H_2_S and NH_4_ gases were either oxidized or lifted to higher levels and exported to Havsten Fjord, and almost 165 tonnes of oxygen were present in the deepwater. About 6 tonnes of PO_4_ remained in the basin. At the same time, salinity increased from 28.7 to 29, temperature from 7.9 to 8.5 °C, and nitrate (NO_3_) from 0 to 15 tonnes—most of this formed from oxidation of NH_4_ via nitrite (NO_2_), see Fig. 4d. All the data from the intensified established monitoring program in the By and Havsten Fjords obtained by BOX can be downloaded from http://produkter.smhi.se/pshark/datamap_bohuskusten.php?language=s.

Time-averaged deepwater salinity (2000–2010) before the pumping started was 30.8 (Fig. 4f). By the end of 2012 the deepwater salinity was 26.8 but a series of water renewals thereafter increased it to about 30 in spring 2013. Pumping down warm summer water increases the volume mean deepwater temperature by up to about 3 °C (Fig. 4f). The pre-pumping amount of H_2_S varied between 0 and 290 tonnes with an average of 120 tonnes, which corresponds to an oxygen debt of about 220 tonnes. During the major part of 2011, there was H_2_S below about 40 m, i.e., well below the nozzle depth. From Dec. 2011 to Sept. 2013, there was no H_2_S in the water (Fig. 4b). The pre-pumping amount of ammonia (NH_4_) varied between 0 and 45 tonnes with an average of about 25 tonnes, which corresponds to an oxygen debt of about 85 tonnes. During the pumping period—during 2010.8–2012.9—NH_4_ was reduced from 22.6 to 0 tonnes, while nitrate (NO_3_) increased from 2 to 11 tonnes. The transient role of nitrite is clearly visible (Fig. 4e). P (PO_4_) was reduced from 15 to 4 tonnes (Fig. 4c) and silicate from slightly below 100 to about 50 tonnes (Fig. 4d).

### Ecological Changes Due to Pumping

Accumulation in the caged mussels or the passive samplers (SPMD and DGT) did not show any significant change before or after pumping either for metals (except for Fe and Mn as mentioned below) (Table S4 in Electronic Supplementary Material) or for the organic pollutants analyzed (Table S5 in Electronic Supplementary Material). Earlier and late results (March 2013) showed that only minor leakage of water-soluble and bioavailable metals and organic compounds has occurred during the experiment. One reason for this state may be that a change of the bottom sediment redox conditions took a long time due to the previous existing heavy debt of oxygen, a main factor regulating any leakage of pollutants from the sediment surface. The surface of the sediments had been oxidized at all depths early in 2012 and was still being oxidized in Sept 2013, about 9 months after the final termination of pumping.

**Fig. 5:**
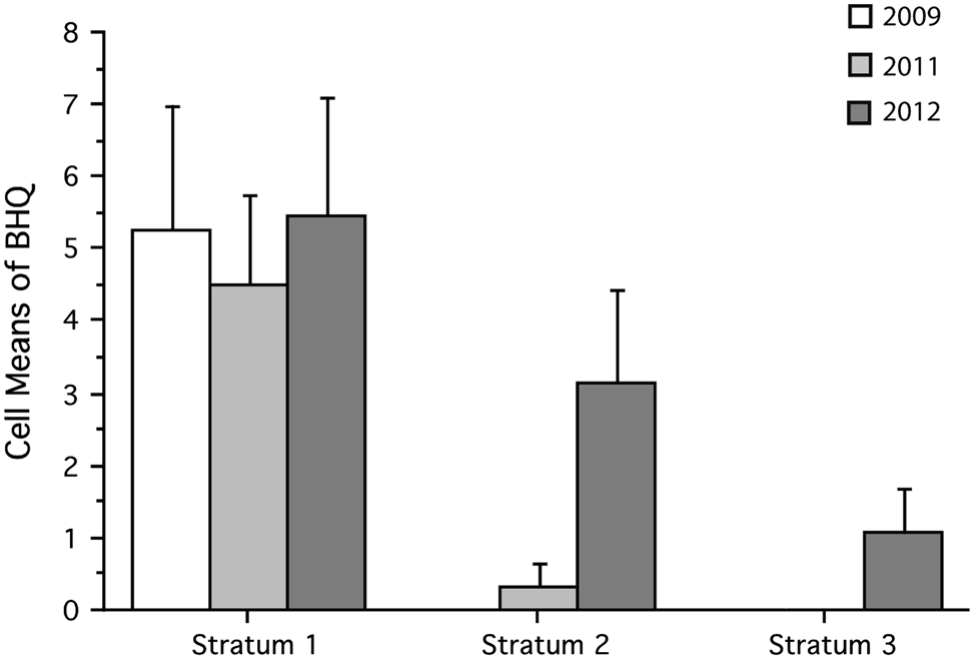
The mean (+95 % confidence interval) of the BHQ index and the interaction between different years and depth strata (10–16, 16–22 and >22-m, respectively) in the By Fjord (*n* = 4)

The photographed sediment profiles indicated changes over time in benthic habitat quality (BHQ) in By Fjord related to the oxygenation of the deepwater. Temporal changes in three different depth strata are described below. Stratum 1 (10–16 m) includes stations at 10-, 12-, 13- and 15-m depths. Of these, only the station at 15 meters, below sill depth, showed a bottom with a very low BHQ, between 0 and 2. BHQ varied between 5.2 and 7.5 for stations shallower than sill depth. The results of a two-factor ANOVA, with “time” and “deep strata” as fixed factors, displayed no significant changes over time in the shallowest depth stratum (Fig. 5).

**Fig. 6:**
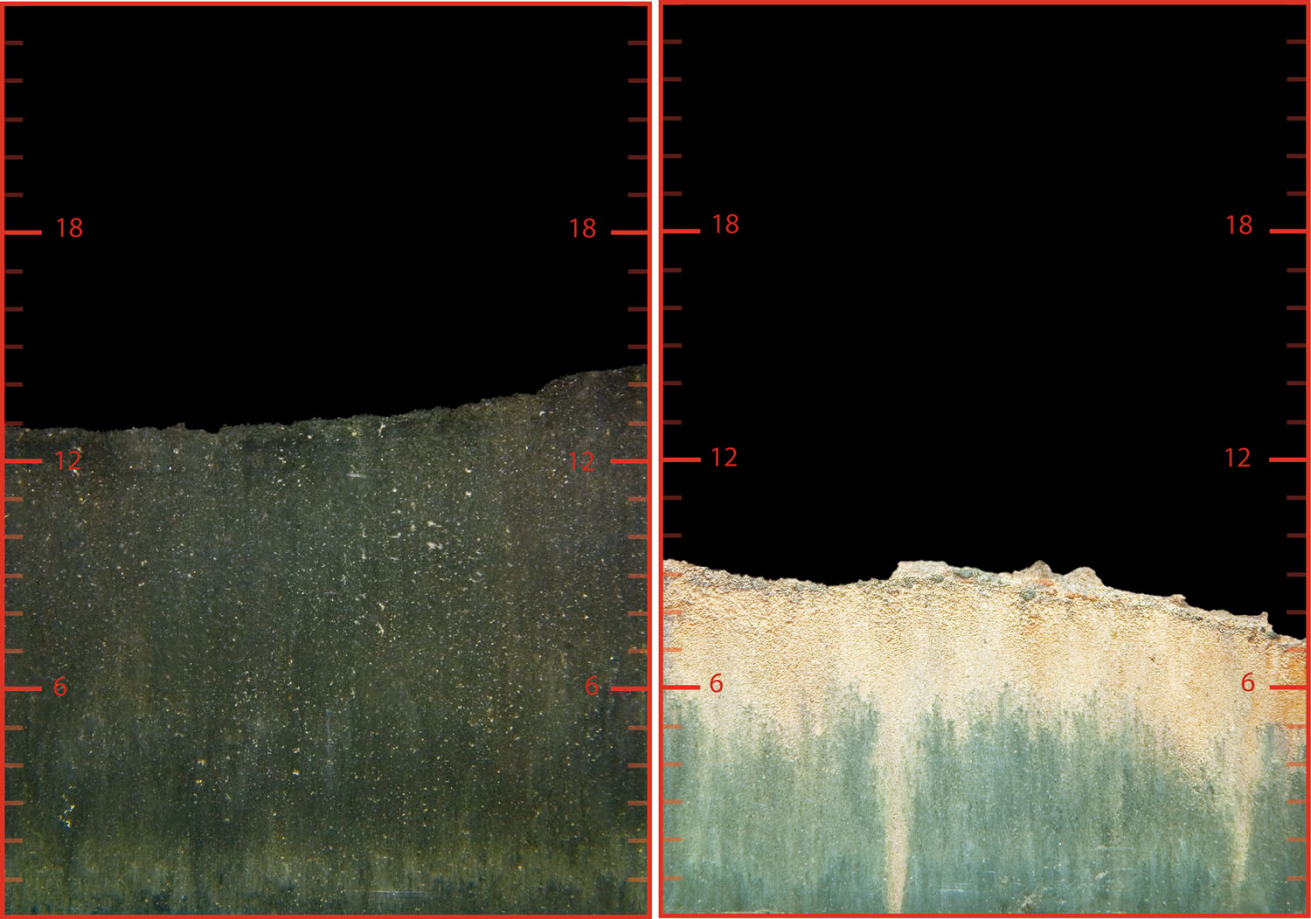
Sediment profile images from station B19 photographed 2009 and 2012. Colors are digitally enhanced, and the scale is in centimetres. An oxygen-reduced sediment bottom, free from macroscopic life, can be seen in the image from 2009 (*left*). The image from March 2012 (*right*) indicates a sediment bottom that has been recolonized and displays a clear light brown oxidized layer, and at least two burrows leading down into the sediment

Stratum 2 (16–22 m) included stations from 17, 18, 19 and 20 meters. All stations are below the sill depth. No macroscopic life was observed at the first sampling in 2009, and BHQ for all the four stations was zero. Tendency to an improved benthic quality was seen during 2011, with a BHQ that varied between zero and one. In some of the images from 2011, bright spots were seen occasionally, which may indicate an early patchy oxygenation of the bottom. However, this was only observed in one out of four pictures in the year 2011 and had no impact on the classification of stratum 2. BHQ increased significantly (*p* = 0.02) in stratum 2 during 2012 compared with the previous years (Fig. 5). The average BHQ increased from 0.3 to 2.4 over a time period of 9 months. This increase mainly depended on the improvement seen at station B18 and B19 (Fig. 6). BHQ increased from 0 to 3.3 ± 2.0 SD at station B18 and from 0 to 6.5 ± 1.0 SD at station B19. Sediment with tube building polychaetes could also be seen at the cameras “mud doors” when it was brought up to the surface, at these stations. Bright spots like those seen at station B20 in 2011 could also be seen in most images from station B17 and B20 from the sampling performed in March 2012. These findings contributed to an increased BHQ in this stratum.

Stratum 3 (>22 m) included stations from 26-, 30-, 38- and 39-m depths. Variance analysis showed that significant changes did occur in the stratum over time since BHQ was significantly (*p* = 0.001) higher in 2012 than that in earlier years (Fig. 5). Bright spots, probably small oxic areas, were seen in several SPI images taken in March 2012. This is also reflected in the average BHQ that increased from 0 to 1.1 ± 1.1 SD.

**Fig. 7:**
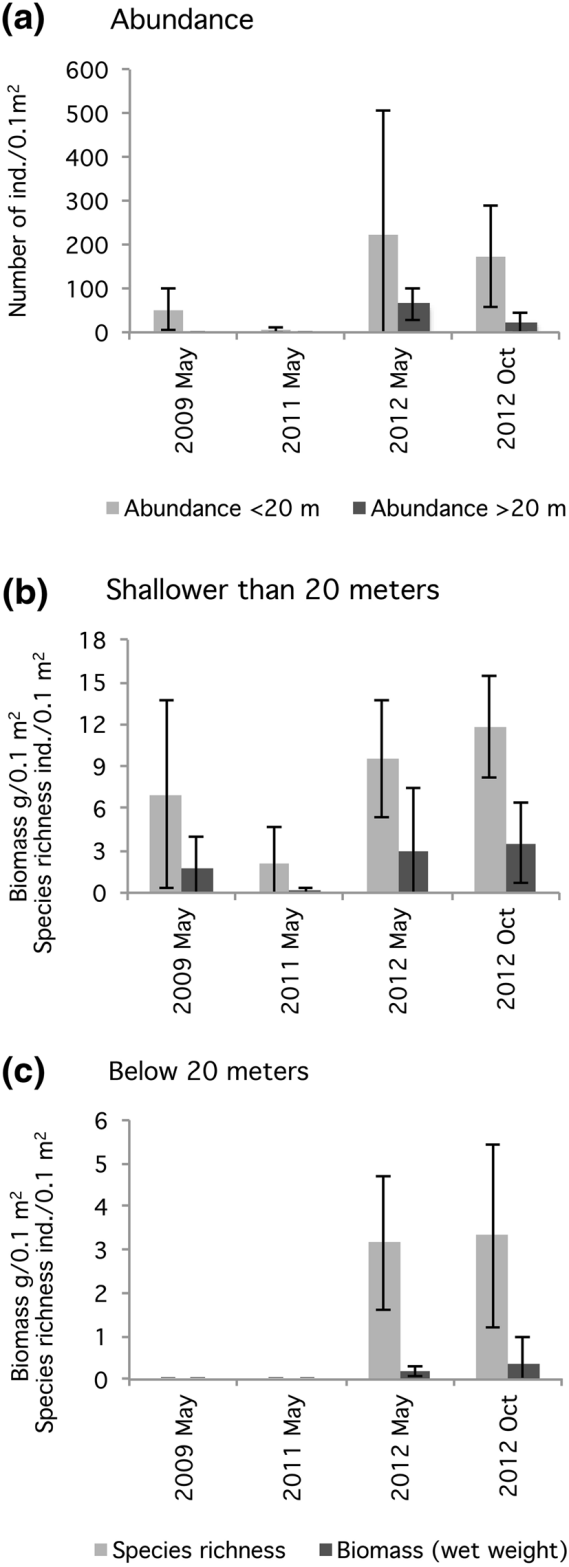
The mean (+95 % confidence interval) abundance of benthic infauna below and above 20-m depth in the By Fjord during 2009–2012 (*upper*). The mean (+95 % confidence interval) species richness and biomass of benthic infauna: above 20-m depth (**b**) and below 20-m depth (**c**) in the By Fjord during 2009–2012

The benthic infauna community showed significant differences in structure over the years. Fauna also colonized the deeper parts of the fjord, down to 40 m, as observed in May 2012. Throughout the study, the species dominating at the different depths changed, and the colonization of the deeper parts consisted of mainly opportunistic species, i.e., *Capitella capitata* (Table 1). Abundance data did not fulfill the requirement of variance homogeneity and were therefore analyzed through Kruskal–Wallis test. The result showed a significant difference between the sampling occasions for both depth intervals (Shallow area, *x* ^2^ = 9.451, df = 3, *p* = 0.024; Deep area, *x* ^2^ = 9.333, df = 2, *p* = 0.009). Follow-up analysis through Multiple Comparison of Means showed a significant difference in the abundance for the deep bottoms between 2011 and May 2012 (*p* = 0.004). For the shallow bottoms, a significant difference in abundance was shown between 2011 and October 2012 (*p* = 0.036). During the studied period, the abundance for deep bottoms peaked with approximately 1500 individuals m^−2^ in May 2012 throughout the fjord (Fig. 7a).

**Table 1:** The most common species on shallow (10–20 m) and deep (>20 m) bottoms are shown. Note that the × in May 2011 for the deep bottoms represents only one species

Dominating species	Shallower than 20 m				Deeper than 20 m			
	2009 May	2011 May	2012 May	2012 Oct	2009 May	2011 May	2012 May	2012 Oct
*Mytilus edulis*	×							
*Phoronis muelleri*	×	×	×	×				
*Chaetozone setosa*	×							
*Scoloplos armiger*		×						
*Varicorbula gibba*		×						
*Capitella capitata*			×			×	×	×
*Trochochaeta multisetosa*			×					
*Heteromastus filiform is*				×				
*Scalibregma inflatum*				×			×	×
*Polydora ciliata*							×	
*Polydora caulleryi*								×
*Peringia ulvae*								×

In 2011, there was a small decrease in the species richness compared to 2009 for shallow bottoms. However, this decrease was only apparent for 2011. During the whole year of 2012, the species richness increased in the fjord. The biomass followed the same pattern as species richness with the highest biomass in October 2012 (Fig. 7b, c).

There was no structural difference in the plankton communities between the By Fjord and the Havsten Fjord from August 2010 to December 2012. This was tested with univariate statistical tests on the following parameters: species number, species richness, biovolumes and ratio autotrophic phytoplankton. Differences in the plankton communities from By Fjord and Havsten Fjord were also compared using multi dimensional scaling (MDS) analyses. No difference could be found between the By Fjord and the Havsten Fjord in the surface water (0–10 m) plankton community (Norén 2012).

**Fig. 8:**
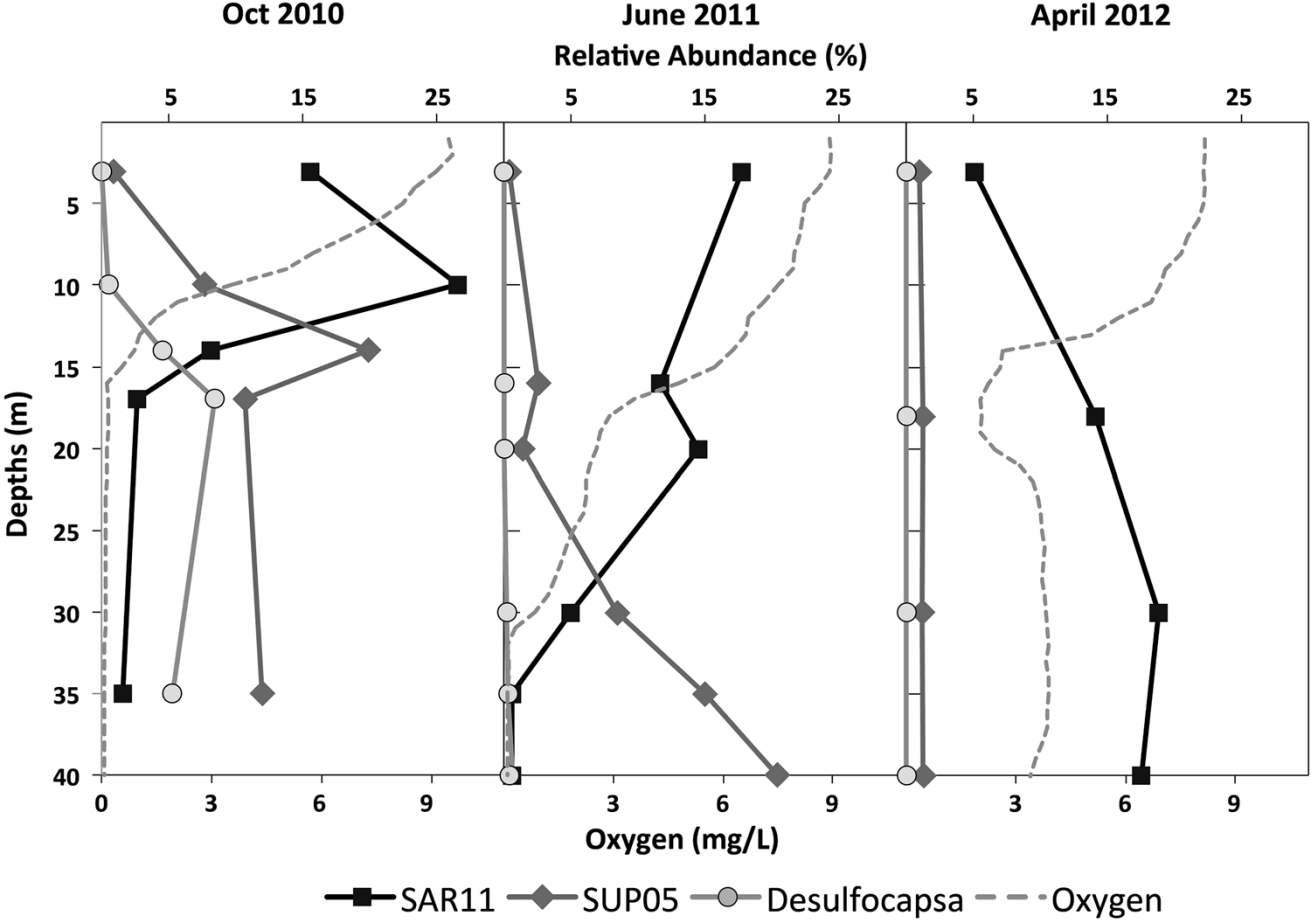
Abundances of SAR11 and SUP05 clade bacteria and Desulfocapsa in the water column of the By Fjord: October 2010 with stratified water column and anoxic bottom waters; June 2011 during oxygenation; and April 2012 after oxygenation

We observed a change in bacterial community structure in the deeper parts of the water column in response to the oxygenation. Before the influx of oxygen-rich water, three distinct bacterial communities could be identified. We observed a surface community above 5-m depth, a chemocline community in samples from 5 to 15 m, and a deep hypoxic community below 15 m where really low oxygen or anoxic conditions occurred. After oxygenation, the chemocline community could be detected down to 30 m, reflecting the novel conditions with oxygen and no sulfide at these depths (Forth et al., in preparation). These findings were confirmed by the distribution of indicator species for the different conditions (Fig. 8). *Desulfocapsa* sp., typically present in sulfidic water (e.g., Finster et al. 1998, 2013), disappeared in the deepwater as soon as oxygen was introduced. SUP05 cluster bacteria, known to thrive under hypoxic and anoxic conditions (e.g., Lavik et al. 2009; Canfield et al. 2010), showed reduced abundance in the lower part of the water column already during oxygenation (June 2011) and disappeared nearly completely by April 2012. In contrast, the aerobic SAR11 clade bacteria could now be detected deeper into the water column. We also found indications that the bacterial community below 15 m became more similar to that in the naturally oxic Havsten Fjord.

### Sediment–Water Fluxes of Phosphorus

Fluxes of DIP across the sediment–water interface were measured in situ using benthic chamber landers both at deep anoxic bottoms and at shallow permanently oxic bottoms. At the deep originally anoxic bottoms, benthic DIP fluxes were measured both before and after engineered oxygenation of the bottom water. The benthic DIP flux was significantly higher at the anoxic than at the permanently oxic stations. Parallel measurements of benthic dissolved inorganic carbon (DIC) fluxes showed that the flux from anoxic bottoms was richer in P than what would be expected from the Redfield C:P ratio (106:1). The C:P ratio of fluxes from oxic bottoms was, in contrast, much higher than the Redfield ratio (Viktorsson et al. 2013b). This suggests that DIP produced in the sediment by degradation of organic matter was to a large extent immobilized in the sediment during oxic conditions in this marine environment. These flux measurements suggest that oxygenation decreased the DIP flux from formerly anoxic By Fjord sediments with a factor of up to about five. Results for anoxic conditions are presented in Viktorsson et al. (2013b).

Estimates of sediment–water fluxes were also made from budget calculations of DIP using hydrographical data for both oxic and anoxic conditions in the water below sill depth. The calculations were done for purely diffusive conditions, i.e., for periods when effects of dilution and pumping vanish. The increase in the rate of DIP in the deepwater during anoxic conditions was much larger than the one would expect from the rate of organic matter degradation, estimated from H_2_S production and Redfield stoichiometry. This excess flux of DIP into the deepwater that continued throughout the first year of oxygenation was most likely due to leakage from the sediments. However, even the deep sediment surfaces were distinctly oxidized in spring 2012 when indeed a yellow and brownish sediment surface could be seen on top of sediment cores (c.f. Fig. 6). Under these conditions, the DIP concentration even decreased in the water column under oxic conditions despite ongoing oxygen consumption. Sediment uptake of P is evident, e.g., during the period from April to June 2013, after the last salty (Fig. 4f) deepwater renewal in the beginning of April 2013 (Fig. 4c). We have thus found from both budget calculations and direct flux measurements using chambers that the benthic DIP flux decreased only when an oxidized top layer had formed on the sediment surface, and not when the bottom water first became oxygenated. The high correlation between O_2_, Fe^2+^, and DIP (Fig. 4a, c) is usually explained by iron’s redox dependence (Mortimer 1941). Particulate FeOOH is formed when dissolved Fe^2+^ is oxidized (Slomp 2011), often through microbial activity (De Schamphelaire et al. 2007). DIP is easily bound to these oxyhydroxides and will coprecipitate with them. This represents an efficient retention mechanism for P. However, this process is reversed when all O_2_ is consumed, and DIP will be mobilized and finally return to the water mass. Further, the similarity in behavior between DIP and DSi in the water column (Fig. 4c) may be a reflection of their common high affinity to Fe-oxyhydroxides (e.g., Mortimer 1941; Cornelis et al. 2011). Mn has similar redox dependence as Fe, but its oxidizing kinetics is much slower, and the existence of several oxidizing states of Mn makes its dynamics more complicated. Nevertheless Mn^2+^ seems well correlated to Fe^2+^ (Table S6 in Electronic Supplementary Material) despite their different redox potentials in this partly hypoxic fjord (Stumm and Morgan 1996). After the first major water exchange, large stocks of Fe^2+^ were only found below the level of the pump outlets (ca 35 m).

## Discussion

The pump system developed by the BOX project is suitable for inshore applications. Obviously, to obtain successful oxygenation and a realistic cost estimate of the pumping, it is paramount to estimate correctly in advance the pumping rate needed to keep the deepwater oxygenated as done for the present case (Stigebrandt and Liljebladh 2011). A description of the near- and far-field flows induced by the pumping will be published elsewhere.

If water exchange occurs when the deepwater in the Havsten Fjord has lower oxygen concentration than the residing deepwater in By Fjord, then oxygen conditions in the By Fjord deepwater deteriorate. This actually happened in October 2011 which can be seen as a relatively warm inflow (Fig. 4a, f). This event underlines that the conditions in neighboring basins must be considered when planning for engineered oxygenation.

Forced oxygenation of the deepwater of the fjord had a significant effect on benthic DIP fluxes from the originally long-term anoxic bottoms. The efflux decreased by up to a factor of about five as a result of the oxygenation (Hall et al. in preparation). This is similar to the decreased efflux of DIP during events of natural oxygenation in the Bornholm Basin (Stigebrandt et al. 2013). Both the direct flux measurements using chambers and the analysis of sediment fluxes using hydrographical data indicate that the decrease in DIP fluxes from the By Fjord sediment coincided with the establishment of an oxidized top layer of the sediment. Hence, oxygenation of only the bottom water was not enough to facilitate P retention in the sediment. It was also necessary to have the surficial sediment oxygenated.

Following the artificial oxygenation of the deeper earlier azoic bottoms of the fjord (Rosenberg 1977), a change in the infaunal community appeared for all the sampled depths. The results showed great differences in the community structure below 20 m, which, together with an increased abundance, demonstrated a colonization of the previously azoic deeper bottoms. An infaunal community was established during 2012. However, the community underwent major changes between the two sampling occasions in May and October, which could be related to seasonal differences in the infaunal community. Results regarding abundance, species richness and biomass followed the pattern expected from a colonization of a previously azoic seabed in accordance with the Pearson–Rosenberg model (Pearson and Rosenberg 1978).

From a stakeholder perspective, this experiment has been successful since local anglers generally believe that the great improvements in fish catches in the By Fjord during the last few years to a large extent are due to the experiment. This is described in several articles in local newspapers (e.g., http://bohuslaningen.se/nyheter/uddevalla/1.2277294-byfjorden-ett-fiskeeldorado). The simplest explanation is that bottom areas and water volumes with acceptable oxygen conditions to be inhabited by fish at least doubled during the experiment. Local authorities now (March 2014) investigate the possibility to build a permanent construction for the engineered oxygenation of the deepwater as decided on October 30, 2013 by the municipal executive board of Uddevalla, (§ 276 in the minutes of the meeting that can be downloaded from http://Uddevalla.se).

## Conclusions

A specific pump system for the By Fjord was constructed for the environmental engineering experiment to oxygenate the deepwater. It pumps oxygen-saturated surface water of low salinity into the deepwater, which then obtains oxygen both from the pumped surface water and from an increased frequency of deepwater renewals by inflowing water from the Havsten Fjord. Most of the deepwater of the By Fjord was kept oxic by pumping from late November 2010 up to December 2011. Pumping continued till December 2012, and the entire deepwater was oxygenated up to as long as August 2013, partly due to the large water exchange in April 2013, facilitated by the deepwater salinity reduction caused by the pumping.

The experiment drastically changed the biogeochemical state of the deepwater of the By Fjord. Hydrogen sulfide was replaced by oxygen. The content of phosphate in the water column decreased radically to typically only 20 % of what it was before the experiment. This was partly due to the increased ventilation and partly due to the strongly reduced leakage from the sediments that occurred when the sediment surface became oxidized. Before the experiment there were large amounts of ammonium in the deepwater, but after oxygenation, nitrate became the dominating DIN component. These changes should imply much reduced ecological stresses on the upper layers of the adjacent Havsten Fjord when these, as a result of deepwater renewal, receive old deepwater from the By Fjord.

Repeated exposure of caged mussels and passive samplers (SPMDs and DGTs) during the oxygenation did not show any increase in the leakage of measured organic pollutants or toxic metals from the bottom sediment. The concentrations of dissolved Fe and Mn in the bottom water followed changes in redox: decreasing or disappearing during oxic conditions, and increasing during reducing conditions.

Analysis of sediment profile images indicated that the benthic habitat quality (BHQ) improved over time in the By Fjord where differences were mainly seen in water depths >16 m. This was also seen in the benthic fauna community. Colonization of benthic fauna was evident during 2012 down to 40-m water depth. An increase in the abundance, species richness, and biomass were evident compared with 2009. The bacterial community was the first to show changes after the oxygenation, with aerobic bacteria also thriving in the deepwater. Oxygenation of the deepwater in the By Fjord did not result in any differences between the phytoplankton communities in the By and Havsten Fjords.

The DIP fluxes from the originally anoxic sediments of the By Fjord became substantially lower after oxygenation of these sediments compared with what they were before oxygenation. This is a unique finding since it is the first time oxygenation via environmental engineering of an anoxic marine basin has been shown to influence benthic P fluxes. It is also the first time an environmental engineering experiment with oxygenation has shown ecological effects like colonization of earlier azoic bottoms.

Making oxygenation experiments in small anoxic basins is a logic way to learn about the environmental impact of forced oxygenation. Many of the environmental effects observed in the By Fjord, e.g., nutrient dynamics, (lack of) leakage of organic pollutants and toxic metals from sediments, and colonization of the sea bed, can be expected to occur also in larger systems like the Baltic proper after switching from anoxic to long-term oxic conditions. It should be possible to verify some results obtained in the By Fjord experiment by studies of basins switching between oxic and anoxic states, like, e.g., phosphorus dynamics in the Bornholm Basin (Stigebrandt et al. 2013). However, several questions regarding the ecological consequences of oxygenation are basin specific, e.g., the cod recruitment in the Baltic, and must be analyzed in its proper context.

## Electronic supplementary material

Below is the link to the electronic supplementary material.

Supplementary material 1 (PDF 162 kb)
