# Supplementary material for: An Experiment with Forced Oxygenation of the Deepwater of the Anoxic By Fjord, Western Sweden
Source: Ambio. 2014 May 1;44(1):42–54. doi: 10.1007/s13280-014-0524-9 (PMC4293361; doi:10.1007/s13280-014-0524-9)
Supplement: Supplementary file 1 — Supplementary material 1 (PDF 162 kb) [file 13280_2014_524_MOESM1_ESM.pdf]

AMBIO

Electronic Supplementary Material

An experiment with forced oxygenation of the deepwater of the anoxic By Fjord, western Sweden

Stigebrandt, A, Liljebladh, B, de Brabandere, L, Forth, M, Granmo, Å, Hall, POJ, Hammar, J, Hansson, D, Kononets, M, Magnusson, M., Norén, F, Rahm, L, Treusch, AH, Viktorsson, L,

S1. Estimate of pumping costs based on experience from the By Fjord Experiment

Below we present the hardware costs (no development fees included) for a pumping system similar to that used by BOX in the By Fjord (Table S1a). We also present approximate annual costs to run the pumping system based on the costs we had in BOX (Table S1b). It is assumed that the pumps are on duty 10 months (7200 h) per year. We have assumed that the life of the pumping system is 10 years. The cost of depreciation is computed assuming 5 % annual interest on capital. Costs for observation of state parameters of the basin where pumping is undertaken are not included. (Conversion rate used in Table S1a and Table S1b: 1 USD = 6.25 SEK).

Table S1a Purchase costs of pumping system

Item	USD
Reinforced concrete floating jetty anchored and ready to use in the By Fjord	31 500
Shore cables, electrical cabinets and installation costs	47 300
2 pumps, each using 12 kW and pumping 1 m^3s^{-1}	39 400
Intake cones, outlet nozzles, feather clutches, fabric pipes	47 300
Others	8 000
SUM	173 500

Table S1b Annual costs to pump

Item	USD $year^{-1}$
Electrical power, 7200 h x 24 kW x 1 SEK h^{-1}	27 700
Maintenance, 5 days: work boat	11 800
Maintenance, 5 days: 2 persons	5 500
Maintenance, spare parts	11 800
Pump manager, 10% of full time	9 500
Depreciation (annual interest rate equals 5 %)	22 100
SUM	88 400

S2. Methods used in the established monitoring programme

List of Methods, SMHI Oceanographic Laboratory

SMHI Prepared 2003-05-07 **Annex I** Quality Manual Revision 2012-03-01 4 pages

Oceanographic Laboratory Approved by CShl DM # 14302:11

**Variable**	**Method**	**Measurement principle**	**Sample Type**	**Uncertainty**	**Range**
Total alkalinity (*)	1	Potentiometric titration	1:3	5%	0.5-3.0 mM
Ammonium-N (¤)	1	Manual spectrophotometry	1:3	14%	0.20-100 µM
Ammonium-N	30	Aut. fluorimetry	1:3	± 7%	0.20 to 10 µM
Depth (pressure) (¤)	2	CTD probe (Sea-Bird)	1:3	± 0.7 dbar [1)]	0.1-6800 dbar
Phosphate-P	1, 3, 4	Flow analysis, spectrometric detection	1:3	12% (0:02 to 0:20)<br>4% (0.20-10)	0.02-10 µM
Phosphorus, Total (*)	1, 3, 5	Persulfate oxidation + flow analysis, spectrometric detection	1:3	18% (0.05-1.0)<br>12% (1.0-10)	0.1-10.0 µM
Chlorophyll *a* (*)	1, 6	Ethanol extraction + fluorimetric detection	1:3	20% (0.1-1.0)<br>12% (1.0-100)	0.1-100 mg / l [2)]
Nitrogen, total (*)	1, 5, 7, 8	Persulfate oxidation + flow analysis, spectrometric detection	1:3	14% (5-100)	5-100 µM
Nitrate-N	1, 7	Flow analysis, spectrometric detection	1:3	7% (0.1-1.5)<br>4% (1.5-50)	0.10-50 µM
Nitrite-N	1, 7	Flow analysis, spectrometric detection	1:3	11% (0.02-0.2)<br>3% (0.2-10.0)	0.02-10 µM
Particulate Org.C (*)	1, 10, 11	Elemental Analyzer	1:3<br>10:4	30%	7-100 µM
Particulate Org. N (*)	1, 10, 11	Elemental Analyzer	1:3,<br>10:4	30%	0.7-20 µM
pH (¤)	1, 12	NBS buffer	1:3	± 0.6%	7-9
Sampling (¤)	6	Water sampling	1:3		
Salinity (*)	1, 13	Conductivity, laboratory salinometer	1:3	± 0:02 units	2-40 (PSS 78)

Salinity (¤)	1, 2<br>22	CTD probe (Sea-Bird)<br>CTD probe (SAIV)	1:3<br>1:3	± 0:01 S / m [1] ±<br>12:02 pts [1]	0.01-7 (Siem / m)<br>0.1-40 (PSS 78)
Silicate	1, 14	Flow analysis, spectrometric detection	1:3	8% (0.1-5.0)<br>3% (5.0-200)	0.1-200 µM
Sulphide	1	Manual spectrophotometry	1:3	9%	1.0-300 µM
Oxygen	1, 9	Iodometric titration	1:3	10%<br>5%	0.02-2 ml / l<br>2-15 ml / l
Temperature (¤)	1, 15	Reversing thermometer	1:1, 3	± 0.02 ° C	-3 - +35 ° C
Temperature (¤)	1, 2<br>22	CTD probe (Sea-Bird)<br>CTD probe (SAIV)	1:3<br>1:3	± 0.01 ° C [1]<br>± 0.01 ° C [1]	-5 - +35 ° C<br>-2 - +40 ° C
Phytoplankton, (sampling and analysis)	6, 16-21	Microscopy	1:3	Cells/L biovol. (mm ^{3})<br>40% (1-99 µm $^{3)}$<br>48% (100-999 µm $^{3)}$<br>53% (1000-9999 µm $^{3)}$<br>42% (10000-99999 µm $^{3)}$<br>29% (> 100,000 µm $^{3)}$	
Primary production with incubator	1, 23-29	^{14}C uptake - CPM measurement	1:3		Production value represented by CPM> 50<br>0.1-50 mgC / h

Methods marked with (*) are performed in the laboratory only, ie. not in field.
Methods marked with (¤) are performed in field only.

[1] Measurement uncertainty according to the manufacturer's calibration data.
[2] Chlorophyll samples with concentration above 25 µg/l are diluted or measured in a spectrophotometer.

Explanations

1 Sample Types

1 Water

1:1 Freshwater / Pool water
1:2 Drinking water
1:3 Seawater / Brackish
1:4 Wastewater / Leachate
1:5 Sludge / Sediment
1:6 Biological material (Biota)
1:10 Other

2 Uncertainty

Expanded measurement uncertainty. The stated uncertainty of the chemical parameters is calculated using a coverage factor of 2, which gives a confidence level of approximately 95%. Measurement uncertainty for phytoplankton is the standard deviation (RSD%) between 4-5 replicates of natural samples.

Range

Range refers to the working concentration range of the analysis. At higher concentrations the samples may be diluted to the working range.

Method References

1. Grasshoff, K., K. Kremling and M. Ehrhardt, eds, (1999) Methods of Seawater Analysis. 3 rd ed. Wiley-VCH.
2. Manual f o r CTD probes: SBE 9/11 plus. (2004) Sea-Bird Electronics, Washington, USA.
3. Manual operation Alpkem. Flow Solution IV. Advanced Technology in Flow Analysis. Orthophosphate in Seawater by Segmented Flow Analysis (2000) OI Analytical Publication 15,050,600th
4. ISO / FDIS 6878th (1998) Water quality - Spectrometric determination of phosphorus using ammonium molybdate.
5. Valderrama, J. (1981) The simultaneous analysis of total nitrogen and total phosphorus in natural waters. Marine Chemistry, 10, 109-122.
6. Manual for Marine Monitoring in the COMBINE Programme of HELCOM http://www.helcom.fi/Monas/CombineManual2/CombineHome.htm
7. Manual operation Alpkem. Flow Solution IV. Advanced Technology in Flow Analysis. Nitrate plus Nitrite Nitrogen and Nitrogen in Seawater by Segmented Flow Analysis (2000) OI Analytical Publication 15,040,900th
8. EN ISO 11905-1. (1998) Water quality - Determination of nitrogen - Part 1: oxidative digestion with peroxodisulfate.
9. SS-A 25 813. (1993) Water Unders island to participate - Best ä mning of content l o st oxygen - iodometric method
10. Operating Manual. Flash EA 1112 Elemental Analyzer ®. (2004) Thermo Electron Corporation.
11. Niuwenhuize, J., YEM Maas, JJ Middelburg. (1994) Rapid analysis of organic carbon and nitrogen in particulate materials. Marine Chemistry 45, 217-224.
12. ISO 10,523th (1994) Water quality - Determination of pH
13. 'Minisal' Model 2100 Salinometer (1986) Operating and Maintenance Manual. AGE.
14. Manual operation Alpkem. Flow Solution IV. Advanced Technology in Flow Analysis. Silica in Seawater by Segmented Flow Analysis. (2000) OI Analytical Publication 15,060,600th
15. Sea Turtle, Version 2.02/3.02. (2003) User's Guide, Ocean Origo AB, Gothenburg.
16. http://www.bi.ku.dk/ioc/ . (IOC taxonomic reference list of toxic plankton algae).
17. Edler, L. 1979. *Recommendations for Marine Biological Studies in the Baltic Sea. Phytoplankton and Chlorophyll.* Baltic Marine Biologists. (Publication No. Fifth).
18. Hallegraeff, GM, Anderson, DM and Cembella, AD 2003. Manual on Harmful Marine Microalgae. UNESCO Publishing, Paris. ISBN 92-3-103871-0.
19. Hällfors, G. Checklist version 28.11.2001.
20. Olenina, I., Hajdu, S., Edler, L., Andersson, A., Wasmund, N., Busch, S., G ö bel, J., Gromisz, S., Huseby, S., Huttunen, M. ., Jaanus, A., Kokkonen, P., Ledaine, I. and Niemkiewicz, E. 2006. Biovolumes and size-classes of phytoplankton in the Baltic Sea. HELCOM Balt. Sea Environ. Proc. No. 106, 144pp.
21. Sournia, A. 1978. Phytoplankton manual. UNESCO Publishing, Paris. ISBN 92-3-101572-9. Utermöhl, H. in 1958. *Zur Vervollkommnung der quantitativen Phytoplankton-Methodik.* Mitt. int. Ver. Theor. Angew. Limnol. *Vol.* 9, pp. 1-38, pl. 1.
22. Manual for STD / CTD - model SD204. SAIV A / S, http:// www.saivas.no
23. Colijn, F. and Edler, L., 1999. Working manual and Supporting papers on the use of a standardized incubator-technique in primary production measurements. HELCOM Annex C 5.

http://sea.helcom.fi/Monas/CombineManual2/PartC/anxc5.html

24. Gargas, E. and Hare, I., 1976. User's Manual for Estimating the Daily Phytoplankton Production measured in Incubator. Contribution from the Water Quality Institute. Danish Academy of Sciences. No.. 2nd
25. Gargas, E., Hare, I., Martens, P. and Edler, L., 1979. Diel changes in phytoplankton photosynthetic efficiency in brackish waters. Marine Biology 52: 113-122
26. Jassby. AD and Platt, T., 1976. Mathematical formulation of the relationship between photosynthesis and light for phytoplankton. Limnology and Oceanography 21 (4), 540-547
27. Kirk, JTO 1996 Light and photosynthesis in aquatic ecosystems, 2nd ed. Cambridge University Press. 509 pp.
28. Mark Ager, S., Technical instructions for navy surplus on gning, 2.4 Prim æ rproduction. Andersen, Mark Ager & Æ rtebjerg (ed.) 2004-11-09
29. Saksha ug, E., Bricaud, A., Dandonneau, Y., Falkowski, PG, Kiefer, DA, Legendre, L., Mo rel, A., Parslow, J. and Takahashi, M. 1997. Parameters of photosynthesis: definitions, theory and interpretation of results. J. Plank. Res. 19: 1637-1670.
30. Kérouel R Aminot A (1997) Fluorometric determination of ammonia in sea and estuarine waters by direct segmented flow analysis Marine Chemistry 57, 265-275

S3. Table S3

Table S3. Organic pollutants and metals measured in caged common mussels and in passive samplers (DGT) in the Byfjord campaign 2009-2012. Stars indicate metals measured in common mussels.

**PAH**	**Organic tin compounds**	
Naphthalene	Monobutyltin	
Acenaphtylene	Dibutyltin	
Acenaphtene	Tributyltin	
Fluorine	Toluene	
Phenantrene	Benzene	
Anthracene	Etylbenzene	
Fluoranthene	sum Xylene	
Benz(a)anthracene	**Metals**	
Chrysene	As*	Fe*
Pyrene	Cd*	Zn*
Benzo(b)fluoranthene	Co*	Ag
Benzo(k)fluoranthene	Cr*	Al
Benzo(a)pyrene	Cu*	Sr
Dibenz(ah)anthracene	Hg*	U
Benzo(ghi)perylene	Mn*	Eu
Indeno(123cd)pyrene	Pb*	Dr
sum 16 EPA-PAH	Ni*	Dy

S4. Table S4

Table S4. Calculated concentrations (µg/L) of free metal ions in Byfjord deep water before and during oxygenation by using passive samplers (DGT), n=3, ± 95% confidence intervals.

Year<br>Depth<br>Temp<br>Hours<br>Metal	 m<br>°C<br>H 	2009<br>38<br>6.1<br>720<br>Mean	 <br><br><br>95% C.I.	2010<br>38<br>5<br>2016<br>Mean	 <br><br><br>95% C.I.	2013<br>23<br>8.35<br>1008<br>Mean	 <br><br><br>95% C.I.	2013<br>38<br>8.35<br>1008<br>Mean	 <br><br><br>95% C.I.
Al	µg/L	2.6	0.6	0.4	0.3	0.3	0.2	0.4	0.1
Cd	µg/L	0.005	0.0	0.023	0.0	0.009	0.0	0.010	0.0
Co	µg/L	0.05	0.0	0.02	0.0	0.01	0.0	0.01	0.0
Cr	µg/L	0.3	2.3	0.01	0.0	<0.03	0.0	<0.03	0.0
Cu	µg/L	0.005	0.0	0.03	0.0	0.09	0.0	0.07	0.0
Fe	µg/L	29.1	3.4	1.2	1.2	0.5	0.1	0.6	0.4
Mn	µg/L	82.2	10.5	1.7	0.7	2.4	0.2	7.1	0.3
Ni	µg/L	0.2	4.5	0.2	0.0	0.2	0.0	0.2	0.0
Pb	µg/L	0.01	0.0	<0.005	0.0	0.004	0.0	0.003	0.0
Sr	µg/L	4.9	0.5	1.7	0.1	2.5	0.4	2.6	0.3
U	µg/L	0.3	15.8	0.3	0.1	0.5	0.1	0.5	0.1
Zn	µg/L	0.3	5.7	0.3	0.1	0.5	0.1	0.5	0.1

S5. Table S5

Table S5. Detected concentrations of organic pollutants and metals in caged common mussels (*Mytilus edulis L.*) in Byfjord before and during oxygenation, n=3, x mean of 10 individuals, ± 95% confidence intervals.

Year		2009 Impact		2009 Reference		2011 Impact		2011 Reference	
**Temp**	**°C**	**6,1° C**		**5° C**		**8,35° C**		**8,35° C**	
**Hours**	**h**	**720 h**		**2016 h**		**1008 h**		**1008 h**	
**PAH**		**Mean**	**95% C.I.**	**Mean**	**95% C.I.**	**Mean**	**95% C.I.**	**Mean**	**95% C.I.**
fluorene	mg/kg w.w	0.001	0.0	<0.001	0.0	0.002	0.0	0.002	0.0
Phenanthrene	mg/kg w.w	0.002	0.0	<0.001	0.0	0.001	0.0	<0.001	0.0
fluoranthene	mg/kg w.w	0.002	0.0	<0.001	0.0	0.001	0.0	<0.001	0.0
benz(b)fluoranthene	mg/kg w.w	0.002	0.0	0.001	0.0	<0.001	0.0	<0.001	0.0
sum 16 EPA-PAH	mg/kg w.w	0.005	0.0	0.007	0.0	0.004	0.0	0.002	0.0
PAH carcinogenic	mg/kg w.w	0.003	0.0	<0.004	0.0	<0.004	0.0	<0.004	0.0
PAH, sum others	mg/kg w.w	0.005	0.0	0.006	0.0	0.004	0.0	0.002	0.0
**Organic tin compounds**		**Mean**	**95% C.I.**	**Mean**	**95% C.I.**	**Mean**	**95% C.I.**	**Mean**	**95% C.I.**
MBT	µg/kg w.w	1.0	0.0	1.7	0.7	3.2	1.7	2.4	0.9
DBT	µg/kg w.w	4.3	0.7	3.3	2.6	2.4	0.6	1.3	0.1
TBT	µg/kg w.w	8.7	1.3	2.7	0.7	7.8	2.5	2.3	0.6
**Metalls**		**Mean**	**95% C.I.**	**Mean**	**95% C.I.**	**Mean**	**95% C.I.**	**Mean**	**95% C.I.**
As	mg/kg d.w	8.1	1.9	9.2	3.0	1.2	0.1	1.2	0.1
Cd	mg/kg d.w	0.9	0.2	0.7	0.1	0.2	0.0	0.2	0.0
Co	mg/kg d.w	0.5	0.2	0.4	0.1	0.1	0.0	0.2	0.0
Cr	mg/kg d.w	0.3	0.0	0.3	0.1	0.1	0.0	0.1	0.0
Cu	mg/kg d.w	6.2	0.6	5.4	0.5	1.2	0.2	1.1	0.2
Hg	mg/kg d.w	0.06	0.0	0.05	0.0	0.01	0.0	0.01	0.0
Mn	mg/kg d.w	5.4	2.1	5.7	2.3	2.5	0.7	1.4	0.2
Ni	mg/kg d.w	0.9	0.3	0.8	0.2	0.2	0.0	0.2	0.0
Pb	mg/kg d.w	0.6	0.2	0.6	0.0	0.1	0.0	0.1	0.0
Zn	mg/kg d.w	114.3	22.3	91.0	5.6	18.6	2.7	17.4	1.0
Fe	mg/kg d.w	92.8	36.6	129.3	72.3	20.8	1.7	20.3	1.6

S6. Table S6

Table S6: Dissolved Fe and Mn (µg L^{-1}) in the deepest parts of the By Fjord below the outlet from the pumps. The sampling interval for each cation DGT measurement is presented for the period June 2010 – November 2012.

Sampling interval	Fe^{2+} 41m (µg L^{-1})	Fe^{2+} 37m (µg L^{-1})	Fe^{2+} 32m (µg L^{-1})	Mn^{2+} 41m (µg L^{-1})	Mn^{2+} 37m (µg L^{-1})	Mn^{2+} 32m (µg L^{-1})
20100614 - 20100621	21.1	14.0	19.3	45.8	56.5	131.8
20101018 -20101107	35.9	34.0	34.2	50.2	45.4	31.6
20101207 -20110301	0.5	0.4	0.4	1.0	0.9	0.9
20110410 -20110526	52.0	66.8	18.0	95.0	147.0	7.3
20110526 - 20110616	64.4	66.8	7.2	150.0	148.5	18.9
20110616 - 20110724	94.8	7.1	1.1	58.2	8.8	8.7
20110724 - 20110902	89.7	18.5	1.8	78.1	135.0	5.2
20110902 - 20111114	79.5	12.8	1.4	56.7	30.8	5.7
20111218 - 20120117	104.0	1.0	1.0	113.0	14.3	4.7
20120117 - 20120306	22.4	1.2	1.0	45.3	135.0	5.8
20120306 - 20120426	0.9	0.9	1.0	76.6	33.1	1.67
20120426 - 20120521	0.5	0.7	0.4	23.8	33.4	12.7
20120521 - 20120618	1.0	0.4	0.4	65.9	49.0	27.9
20120618 - 20120816	1.5	0.4	0.4	6.0	41.0	25.0
20120816 - 20120830	84.4	1.2	0.6	44.1	22.1	9.6
20120830 - 20121017	128.0	2.1	0.9	11.1	15.2	4.8
20121017 - 20121119	0.2	0.5	0.9	131.0	31.5	5.6
